# SMITracker: An Interactive Platform for Tracking and Analysis of Single-Molecule Interactions with Linear Substrates

**DOI:** 10.34133/csbj.0014

**Published:** 2026-03-18

**Authors:** Arash Ahmadi, Magnar Bjørås, Bjørn Dalhus

**Affiliations:** ^1^ Centre for Computational and Data Science (dScience), Faculty of Mathematics and Natural Sciences, University of Oslo, N-0316 Oslo, Norway.; ^2^ Department of Clinical and Molecular Medicine, Faculty of Medicine and Health Sciences, Norwegian University of Science and Technology (NTNU), N-7491 Trondheim, Norway.; ^3^ Centre for Embryology and Healthy Development (CRESCO), University of Oslo, N-0373 Oslo, Norway.; ^4^ Department of Microbiology, Oslo University Hospital HF, Rikshospitalet and University of Oslo, N-0424 Oslo, Norway.; ^5^ Department of Medical Biochemistry, Institute for Clinical Medicine, University of Oslo, N-0424 Oslo, Norway.

## Abstract

Understanding the interactions of proteins with macromolecular substrates such as DNA, microtubules, and actin filaments is critically important in molecular biology and beyond. Single-molecule experiments provide unprecedented insight into the dynamics of these interactions. However, efficient analysis of large datasets generated by such experiments remains a major challenge. Specifically, in single-molecule experiments that involve scanning of such linear substrates, the absence of a comprehensive and effective system for detecting interactions among millions of frames of high-frequency imaging data and excluding noise without bias hampers research productivity and data quality. To address these limitations, we introduce “Single-Molecule Interaction Tracker (SMITracker)”, an interactive analysis platform designed to enable efficient, accurate, and scalable detection of single-molecule interaction events. The protocol begins with raw image data obtained from single-molecule experiments, which undergoes preprocessing and is transformed into a structured dataset, suitable for processing in R. Using an automatic trajectory detection algorithm and a uniform noise exclusion model, the target data are detected and organized for further analysis. Final outputs include comprehensive diffusion analysis and informative visualization, enabling side-by-side comparison of different proteins and/or experimental conditions. SMITracker is available as an R package for R users and as a Docker image for non-R users, offering a highly effective and convenient solution to the common challenges in analyzing data from single-molecule experiments.

## Introduction

In recent years, single-molecule experiments have become instrumental in understanding the underlying molecular processes of various biological phenomena. Examples include the thermally driven scanning of DNA by proteins or movement of adenosine triphosphate (ATP)-driven proteins on DNA, microtubules, or actin filaments. A typical experimental setup for investigating interactions of proteins with linear substrates involves movement of fluorescently labeled proteins along a substrate held in an elongated form. A multitude of critical insight can be gained from the analysis of such movements, for example, revealing the effect of particular structural elements on scanning [1–4], detecting of modality in scanning [1,5,6], determining the effect of various biochemical environments on the movement of a protein along the substrate [7,8], and observing the interactions of multiple proteins on a single substrate [9,10]. Recent advances in microscopy and experimental methods have made data collection from such experiments more feasible than ever before. However, efficient analytical tools to handle and understand these data types and to extract insights effectively remain underdeveloped.

The target data for single-molecule scanning typically consist of isolated interactions of a labeled protein molecule with a substrate, involving either thermally driven or ATP-driven movements. The datasets from single-molecule scanning experiments tend to contain large numbers of frames with sparsely occurring interactions, and they usually carry a substantial amount of noise. Detecting thousands of these interactions among millions of collected frames, excluding the noise from the data, and gaining insight from analysis of such data can be very time and resource consuming. Previously, we have developed an analytical approach where raw data from single-molecule DNA scanning were analyzed and yielded a range of insightful findings [1,3]. However, using this approach requires users to have a deep understanding of programming in R as well as the underlying physics of the scanning phenomena. For many researchers and domain experts, understanding and utilizing thousands of lines of code can pose a substantial barrier.

To address this challenge, we have developed an interactive R Shiny application called “Single-Molecule Interaction Tracker (SMITracker)”, building upon our prior approach [1,3,11,12], making it more accessible and user-friendly. Users can operate the platform and utilize all its functionalities through a user-friendly interface, including the detection of interactions between fluorescently labeled proteins and linear substrates, performing uniform noise exclusion and in-depth diffusion analysis. Another typical challenge in single-molecule scanning experiments is the difficulty of rapidly obtaining an overview of the data being collected during an experiment. SMITracker enables users to explore and summarize newly acquired datasets within minutes, providing rapid, informative feedback in a laboratory setting. This allows experimentalists to assess data quantity and quality early and to adjust experimental conditions efficiently when needed. The individual SMITracker modules were successfully used to process, analyze, and present data in our single-molecule studies of several DNA repair proteins [1,3].

## Materials and Methods

### Experimental design of single-molecule studies

Typical experiments for producing single-molecule linear scanning data involve movement of fluorescently labeled proteins along a linearly fixed substrate. The movement of the proteins can be either thermally [1,3,13,14] or ATP/energy [9,10] driven, and proteins can be labeled using small organic dyes [1,7,15] or quantum dots [10,14,16]. The substrate for such experiments can typically be DNA [1], microtubule [17], or actin filaments [18]. To observe the relative movement of the target proteins along their respective substrate, the substrate needs to be held in a linear form. Elongation of the substrate can be achieved by different methods including using optical traps [1,18], DNA curtains [19], DNA tightrope [20], or flow [7]. The data are collected as sequences of images in which movement of the fluorescently labeled proteins can be traced.

Our experimental setup for acquiring single-molecule scanning of DNA has been described in detail previously [1,3,21]. Briefly, in this setup, the surface of a coverslip is passivated using polyethylene glycol molecules, with a low proportion of those molecules being biotinylated, which are later associated with streptavidin. This coverslip is used for constructing a flow chamber, and using a microfluidic flow system, the DNA is delivered to the point of observation and bound to the coverslip surface at one end (using a biotin–streptavidin–biotin linkage). The other end of the DNA is later attached to a polystyrene bead, using a digoxigenin–anti-digoxigenin linkage. By holding the bead in an optical trap that is steered using a spatial light modulator (SLM), DNA is elongated to around 95% of its contour length. DNA repair proteins are labeled with fluorescent dye ATTO-647N using a cysteine–maleimide linkage. The labeled proteins are delivered to the point where an elongated DNA molecule is under observation. The sample is illuminated using a highly inclined and laminated optical sheet (HILO), and random associations and scanning of DNA by labeled proteins are observed and recorded using an electron-multiplying CCD camera.

### Development of the SMITracker platform

Due to the stochastic nature of molecular interactions in single-molecule experiments, the acquired data can become extremely large (several million frames) with interactions randomly distributed over time. The first step in analyzing single-molecule scanning data is to locate spatiotemporal coordinates of these interactions in the positional time series datasets. Next, noise from various sources must be uniformly and systematically identified and excluded from the detected trajectories. The platform was developed to address these challenges through independent modules each tackling different aspects of the problem [1,3,11,12]. These modules were later integrated into SMITracker, which consists of a sequence of interactive user interface modules starting with data import and transformation and concluding with plots and export of processed data that reveal insightful information about scanning of linear substrates such as DNA.

### Software implementation

The main code was developed in R and organized into 21 functions, documented using Roxygen2 style [22]. Additionally, one of the more computationally intensive functions was written in C++ and is called from R using Rccp package [23]. Building upon these functions, the interactive platform was developed using Shiny [24]. The user interface was built using UI function, with the backend scripted inside a server function. For enhanced performance stability, the source code was transformed into an R package using the Golem framework [25]. Upon installation and loading of the package, the run_app() function will be exported to the namespace and running this function will initialize the interface. For the C++ function to work properly, RTools [26] should be installed on the system before installing and running SMITracker package. Moreover, to ensure further stability and consistency across environments, we have provided the essential elements as well as the docker file to create docker image for running the platform as a standalone application. This allows non-R users to utilize SMITracker and all its functionalities without needing to use R, which eliminates issues related to dependencies, RTools, or required packages, as everything is provided within the Docker image. The source code and installation instructions for both the R package and Docker alternatives are available on GitHub and Zenodo as a versioned release (v1.0.0) [27]. All analyses reported here were performed using SMITracker v1.0.0 on Windows 10/11 with an Intel Core i5-8500 processor (3.00 GHz) and 16 GB RAM. Note that preprocessing requires Fiji [28] and ThunderSTORM plugins [29].

## Workflow

**Fig. 1.**
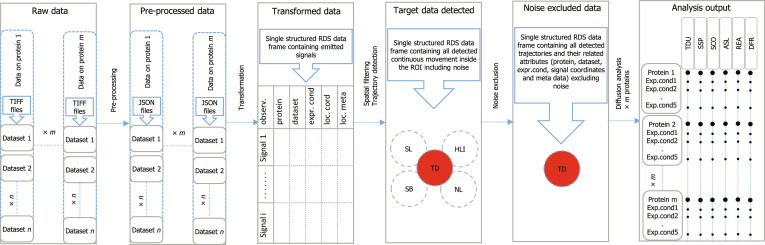
A depiction of the analysis workflow. Panel 1: Structure of raw data for *m* number of proteins each having *n* number of datasets (TIFF files). Panel 2: Structure of preprocessed data where one JSON file is produced corresponding to each TIFF file in the raw data. Panel 3: Structure of the transformed data containing *i* number of localized signals; column names observ., exper.cond, loc.cord, and loc.meta stand for observations, experimental conditions, localization coordinates, and localization metadata (including signal intensity), respectively. Panel 4: Structure of the data after spatial filtering and trajectory detection operations; abbreviations TD, SL, HLI, SB, and NL stand for target data, short-lived noise, high/low-intensity noise, surface-bound noise, and nonlinear movements, respectively. Panel 5: Noise-excluded target data detected along with all localization information and metadata. Panel 6: Output of the overall analysis, sorted for different proteins and experimental conditions; abbreviations TDU, SSP, SCO, ASL, REA, and DFR stand for trajectory duration, scanning speed, scanning covering range, accumulative scanning length, redundancy–efficiency analysis, and diffusion rate analysis, respectively.

The use of SMITracker involves several main stages (Fig. 1), each stage described below, with detailed step-by-step instructions given in the Supplementary Materials. The first stage involves preprocessing of the raw data in Fiji. This is followed by 5 stages of data transformation and analysis within the first 5 modules of SMITracker. The final stage involves visualizing the analysis results in the last module of SMITracker.

### Preprocessing of raw data

As shown in the first panel of Fig. 1, the single-molecule data for each protein consist of several TIFF files, referred to as datasets. Each dataset contains multiple frames collected to capture protein–DNA interactions. The light received from the excitation of single fluorophores forms signals, which can originate from freely floating proteins (or unincorporated dyes), surface-bound proteins (or unincorporated dyes), or proteins bound to the linear substrate of interest. In the first step, we need to localize all existing signals, regardless of their state, and record accurate *x* and *y* coordinates as well as signal intensity. The ThunderSTORM plugin in Fiji accurately and reliably performs this operation by fitting a centroid on each signal. This constitutes the preprocessing step (transition from the first to the second panel of Fig. 1), where all existing signals in each dataset are localized and reported in the corresponding JSON files that can be processed by R. The user can choose to localize the signals using the plugin’s user interface in Fiji, or use a script provided on GitHub and Zenodo [27] for automatic preprocessing according to the prescribed procedure.

### Data loading module

The semistructured JSON files produced as a result of the signal localization process should be transformed into structured data-frame formats for further processing and analysis in R. The process of importing and transforming JSON files takes place automatically in the first module of SMITracker (transition between the second and third panel of Fig. 1). In this module, the user provides a set of metadata for each dataset via the file name. The output of this step is a single structured dataset where all information related to the localized signals are organized along with the provided metadata. Each row in the dataset represents a unique observation (or localized signal), and columns are information associated with each observation such as protein, dataset name or experimental condition, spatiotemporal coordinates, and intensity of the signal and the detailed output of the localization process.

### Spatial filtering module

In scanning experiments where the linear substrate is not fluorescently labeled, accurately localizing the substrate is a common challenge. To ensure that all parts of the substrate are included in the acquired data, a larger field of view is captured during data collection. However, the target data are confined to a much smaller field of view where the elongated substrate lies. The spatial filtering module (transition between the third and fourth panel of Fig. 1) allows users to visualize the localized signal from each dataset separately and select the area where the traces of proteins scanning the substrate are located. By excluding nontarget data points, the volume of the datasets is reduced, considerably increasing the efficiency of the trajectory detection module and saving considerable computation time. In addition to the target scanning data, the user needs to select a few points in each dataset where the signals come from a surface-bound protein or dye. These data points define a reference for surface-associated signals to optimize the parameters used by the noise exclusion model. If more than one substrate is attached to a single bead (examples can be seen in the third column of Fig. S1), the traces of proteins along different paths can still be resolved in space and time if the substrate molecules are properly elongated and physically separated. To extract data from such complex datasets, we have provided the users with a tool to split such data into 2 different parts and run the trajectory detection algorithm separately on each part.

### Trajectory detection module

After all datasets are spatially filtered, the next step is to detect the spatiotemporal coordinates of trajectories and organize them for noise exclusion and subsequent diffusion analysis. Trajectories or interactions are defined as continuous positional time series of single molecules scanning along the substrate. The trajectory detection module of SMITracker includes an automatic trajectory detection algorithm as well as a systematic arrangement and preparation of the data for further analysis (transition between third and fourth panel of Fig. 1).

The trajectory detection algorithm employs a frame-by-frame proximity-based tracking approach to identify and reconstruct single-molecule trajectories from localized fluorescent signals. At first, raw localization data in JSON format are converted to long-form tabular format containing frame numbers and *x*–*y* coordinates for each detected signal. The data are then transformed into a matrix representation via the MakeMatrix() function. In this matrix structure, each row corresponds to a unique frame number, while columns represent simultaneously detected signals within that frame. This transformation facilitates an efficient frame-to-frame signal tracking. The core trajectory detection is performed by the FindTrajectory() function, which implements a nearest-neighbor linking algorithm. For each signal in frame *i*, the algorithm searches for the nearest corresponding signal in frame *i* + 1 using Euclidean distance minimization in both *x* and *y* dimensions. A signal is linked to the trajectory only if (a) the displacement along the substrate (Δ*x*) satisfies *dx*_min_ < Δ*x* < *dx*_max_, and (b) the displacement perpendicular to the substrate (Δ*y*) satisfies Δ*y* < *dy*_max_, where d*x*_max_ and *dy*_max_ are user-defined maximum displacement thresholds, and *dx*_min_ (0.0001 nm) prevents erroneous self-linking. Surface-bound signals are detected with more restrictive displacement thresholds (where both *dx*_max_ and *dy*_max_ are set equally, proportional to the localization precision) to identify immobile fluorophores. To initialize this algorithm, the user needs to select a few trajectories and determine initial estimates for *dx*_max_, *dy*_max_, and localization precision (according to procedures explained in the Supplementary Materials).

The algorithm includes separate logics to handle events such as consecutive but distinct trajectories, detection of multiple signals within an acceptable displacement range, and blinking of fluorophores. If a signal at frame *i* represents a potential link but the previous signal at frame *i* − 1 exceeded the displacement thresholds, the previous position is nullified to ensure proper trajectory segmentation. On the other hand, when multiple signals in frame *i* + 1 fall within the acceptable displacement range, the algorithm selects the signal with minimum Euclidean distance, ensuring one-to-one frame-to-frame correspondence. Fluorescent dye blinking events, which create apparent trajectory breaks, are corrected through the CorrectBlinking() function. When a signal is absent in frame *i* but present in both frames *i* − 1 and *i* + 1, and the displacement between these signals satisfies a specified distance criteria (2 × *dx*_max_), the algorithm interpolates the missing position as the mean of the neighboring coordinates, effectively bridging single-frame gaps in trajectories.

At the end, detected trajectories undergo a final processing through ArrangeData(), where they are assigned unique numerical identifiers, sorted by frame number, and joined by all signal localization attributes (intensity, uncertainty, sigma, background) and experimental metadata. At this stage, nontrajectory signals (unconnected localizations) are removed from the dataset. The output of this stage is a single dataset where all potential movements along and around the substrate are detected, properly labeled and organized. These movements might include proteins scanning the linear substrate or can originate from various sources of noise (fourth panel of Fig. 1), which needs to be excluded in the Noise Exclusion module as explained below.

### Visual Inspection module

Due to the illumination of the entire field of view, single-molecule experiments often carry a large amount of noise that can pass through trajectory detection as interaction data points. To address this challenge, the noise exclusion framework employs an adaptive classification approach where parameters of the model are set according to a few representative trajectories for each dataset. A few longest-ranging trajectories for each dataset are selected as representatives, as these are most likely to represent scanning interactions rather than noise artifacts. Depending on the total number of trajectories per dataset and the quality of the data, users can decide how many trajectories are needed for optimizing the parameters of the model (the default is set to 5 trajectories per dataset). In the Visual Inspection module, the interface displays complete *x*–*y* paths of the selected trajectory, along with temporal *x* and *y* progression with intensity profile. The user can remove the data for trajectories that are either flagged for extreme values of intensity or their spatial path does not fit with linear scanning of substrate. Importantly, trajectories excluded at this stage are not manually removed from the dataset; they are omitted only from the set of representative trajectories used for parameter optimization.

### Noise Exclusion module

Noise Exclusion module handles 4 major classes of noise: (a) short-lived detections, (b) surface-bound emitters, (c) nonlinear movement, and (d) intensity-based artifacts. Noise exclusion is implemented using a heuristic-based framework that classifies detected trajectories as genuine signals or noise based on dataset-specific statistical parameters.

Short-lived noise is referred to transient signals arising from freely diffusing labeled proteins or dissociated fluorophores that are identified based on trajectory duration. The AddDurationFilter() function calculates the number of consecutive frames for each trajectory and applies a minimum threshold (minFrameDuration parameter). The value for this threshold can be empirically defined by the user depending on the temporal resolution of the experiment and binding lifetime of the protein, and is applied globally across different datasets.

Surface-bound noise comes from fluorophores that are nonspecifically adsorbed to the coverslip surface and are distinguished by their lack of directional movement. Surface-bound signals, previously identified during the spatial filtering (in spatial filtering module) and detected using restrictive displacement thresholds (2× localization precision in trajectory detection module), serve as a reference for establishing a mobility benchmark for immobile emitters. For each trajectory, 4 positional metrics are calculated:

$$m_{j}\in \left\{{\mathrm{SD}}_{x},\;{\mathrm{SD}}_{y},\;{\text{Range}}_{x},\;{\text{Range}}_{y}\right\},\;j=1,\ldots ,4$$ (1)

where SD and Range denote coordinate’s standard deviation and range in both *x* and *y*. Let $m_{i,j}$ denote metric $j\left(j=1,\ldots ,\;4\right)$ computed from representative trajectory $i\left(i=1,\ldots ,\;N\right)$. For each metrics j, dataset-specific mobility thresholds ($T_{j}$) are derived as:

$$T_{j}={\bar{m}}_{j}+\text{gauge}\times \sqrt{\frac{1}{N-1}\;\sum_{i=1}^{N}{\left(m_{i,j}-{\bar{m}}_{j}\right)}^{2}}$$ (2)

where ${\bar{m}}_{j}$ is the mean of the metric across representative trajectories defined as $\frac{1}{N}\;\sum_{i=1}^{N}m_{i,j}$ and gauge is the user-defined stringency parameter. Each trajectory k is then evaluated by comparing its metric values $m_{k,j}$ to its corresponding thresholds $T_{j}$. Trajectories exceeding a threshold in any metric are classified as mobile, whereas those remaining below all thresholds are classified as surface-bound noise. This dual-metric approach (SD and Range) provides complementary assessments of mobility, where standard deviation captures overall positional dispersion, while range detects even minimal directional drift that might be missed by variance-based measures alone.

Nonlinear movement noise originates from proteins interacting with nonlinearized or freely diffusing substrate fragments, resulting in multidirectional motion. This noise class is identified using perpendicular (*y*-axis) displacement analysis relative to the primary scanning direction. Three perpendicular mobility metrics (${\mathrm{SD}}_{y},{\text{Range}}_{y},{\text{Mean}}_{y}$) are computed. Visually validated representative trajectories (in Visual Inspection module) are used as reference data. For each dataset, thresholds are derived using the same adaptive formulation as above. Trajectories must satisfy all 3 criteria to be classified as genuine; failure of any criterion indicates excessive lateral motion and results in exclusion as nonlinear movement noise.

Intensity-based noise includes both high-intensity fluorophore aggregates and low-intensity autofluorescent background. Mean signal intensity for each trajectory (${\text{Mean}}_{\text{intensity}}$) is used as the intensity metric to evaluate the data compared to values derived from visually validated representative trajectories (in Visual Inspection module). An adaptive threshold (with a similar logic as above) defines the acceptable intensity range for each dataset, and trajectories outside this range are classified as high/low-intensity noise.

All thresholds are calculated independently for each dataset to account for variations in signal-to-noise ratio. While users may adjust gauge parameters after visual inspection, these adjustments are applied globally to entire datasets to avoid dataset-specific bias introduced by users. The resulting noise-filtered trajectories provide a high-confidence dataset suitable for downstream spatial and diffusion analyses of single-molecule interactions.

### Analysis Output module

The process up to module 6 can be applied to data from different experiments, with the results saved in separate output files. As depicted in the sixth panel of Fig. 1, different datasets from multiple experiments can be uploaded sequentially and sorted by protein and experimental conditions. The Analysis Output module is divided into a data overview section (Fig. S7) as well as 6 separate sections each exploring a parameter extracted from the organized trajectory data to describe and characterize the movement of the proteins along the substrate. In each section, the average values of a specific parameter along with their corresponding distributions are visualized for each protein and experimental condition. Users can choose which proteins or experimental conditions to include in the visualizations, allowing for their side-by-side comparison in terms of those parameters. The visualizations provided in this module are intended primarily for exploratory data analysis. However, the tabular data underlying each plot can be exported through the interface, allowing users to perform targeted, hypothesis-driven statistical analyses. The logic behind the extracted parameters is described in the following section, together with representative visualizations generated using data from the Analysis Output module.

## Results

### Intermediate processed data and organized trajectory data

**Fig. 2.**
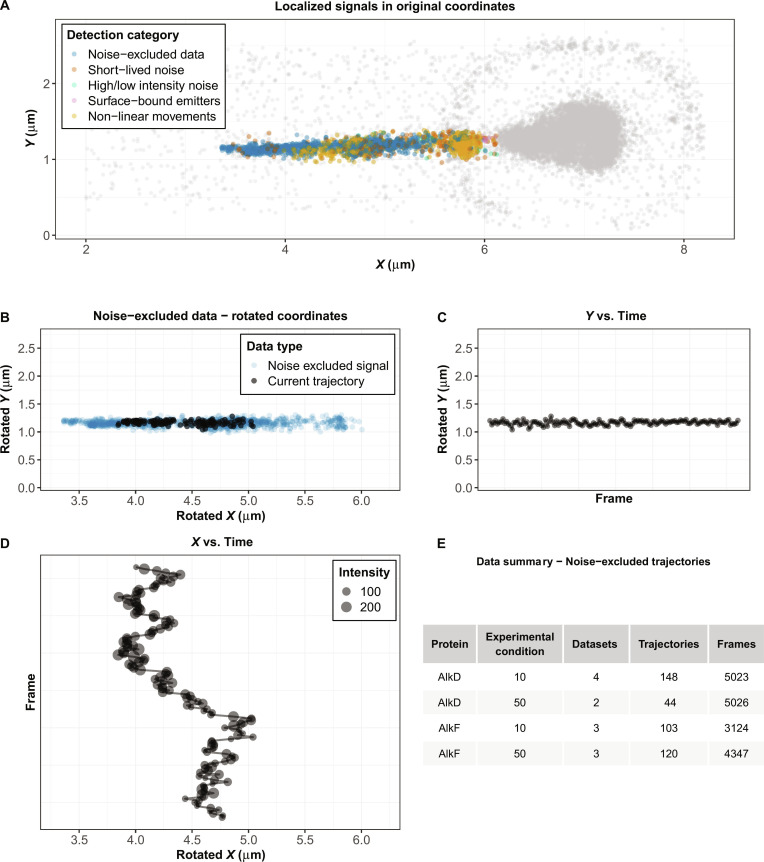
Visualization and inspection of noise-excluded trajectories in SMITracker. (A) Distribution of detected trajectories shown in the context of the full dataset, including noise-excluded signals and different noise classes. (B) Complete *x*–*y* paths of an individual noise-excluded trajectory displayed along with the entire noise-excluded trajectories. Coordinates are rotated to align protein movement along the substrate with the *x* direction. (C) Temporal progression of *y*-coordinate position for a selected trajectory. (D) Temporal progression of *x*-coordinate position and corresponding intensity profile mapped onto the datapoint size. (E) Summary table displaying the accumulation of analyzed data across loaded datasets, including the number of datasets, trajectories, and frames for each protein and experimental condition (i.e., buffer salt concentration in mM).

The first 5 modules of SMITracker each produce an intermediate structured dataset that can be either passed directly to the subsequent module or used independently for downstream analysis. The output of the Noise Exclusion module represents the primary result of the SMITracker workflow, which is a tidy, noise-filtered set of trajectories containing signal coordinates and intensities, organized by unique trajectory identifiers and ready for further targeted analysis. Users can inspect the distribution of noise-excluded trajectories in the context of the full dataset, alongside the excluded noise classes (Fig. 2A). Individual trajectories can also be examined in detail, including their complete *x*–*y* paths overlaid on the entire noise-excluded trajectories (Fig. 2B), temporal *y* progression (Fig. 2C), and temporal *x* progression with corresponding intensity profiles (Fig. 2D). The first section of the Analysis Output module provides an overview of the accumulated data across all loaded experiments (Fig. 2E). This summary table reports key metrics such as the number of datasets, trajectories, and observations for each protein and experimental condition. Individual datasets may contain multiple proteins and/or experimental conditions, and data for a given protein may span multiple datasets. As long as each protein is assigned a unique identifier, its associated trajectories are automatically grouped and analyzed together. The datasets shown in Results are exemplary and are intended to demonstrate the functionality of the interface; they are not meant to provide an exhaustive analysis of these proteins, which has been reported elsewhere [3].

### Scanning speed and lifetime

**Fig. 3.**
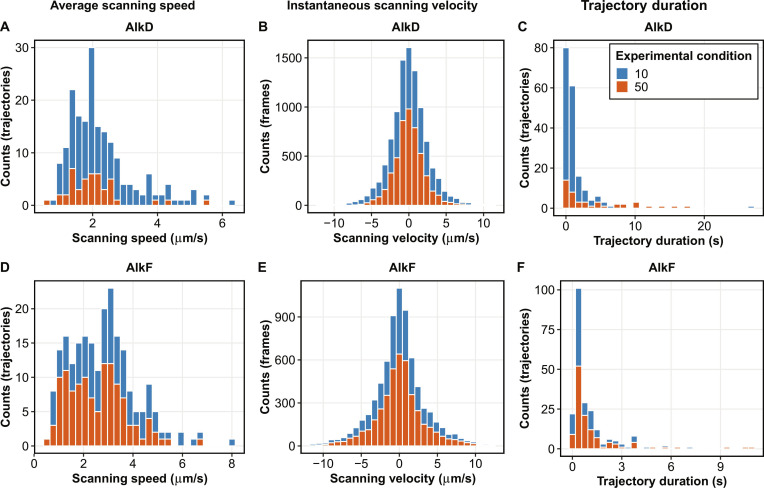
Quantification of scanning speed and trajectory duration using SMITracker. The plots are generated from data obtained from the first 2 sections of the Analysis Output module. Data are shown for 2 proteins (AlkD and AlkF, as indicated in the plots), with histograms color-coded by experimental condition (salt concentration in mM). (A and D) Distributions of trajectories’ average scanning speed. (B and E) Distributions of instantaneous scanning velocity. (C and F) Distributions of trajectory duration. The data shown here for different protein and experimental conditions are exemplary and intended to demonstrate the concepts used in the Analysis Output module.

The value of average scanning speed for each trajectory is calculated as the mean absolute value of the frame-to-frame displacements for each trajectory divided by the frame interval for that trajectory. The distributions of trajectories’ average scanning speed for combination of protein and experimental condition are shown in Fig. 3A and D. Instantaneous scanning velocity is defined as the frame-to-frame displacement of the protein per unit of time. As shown in Fig. 3B and E, the symmetry in the instantaneous scanning velocity plots indicates bidirectional scanning, which is a common characteristic among thermally driven molecules on a linear substrate.

Trajectory duration is defined as the time span of a continuous signal detected from the protein while interacting with the substrate; it starts with the random association of the protein to the substrate, followed by scanning, and ends with discontinuation of the signal when the protein dissociates from the substrate. Trajectory duration is calculated for each trajectory, and its distribution can be reported for each protein/experimental condition combination by selecting the respective options in the interface (Fig. 3C and F). Care must be taken when interpreting the trajectory duration as the true protein binding lifetime, since this value can be affected by longevity of the fluorescent signal (fluorescent lifetime). It is advisable to conduct control experiments where the fluorescent lifetime is measured under similar chemical environment and laser power. Trajectory duration can be considered equivalent to protein binding lifetime if the fluorescent lifetime is consistently longer than the observed trajectory duration.

### Scanning coverage and accumulative length

**Fig. 4.**
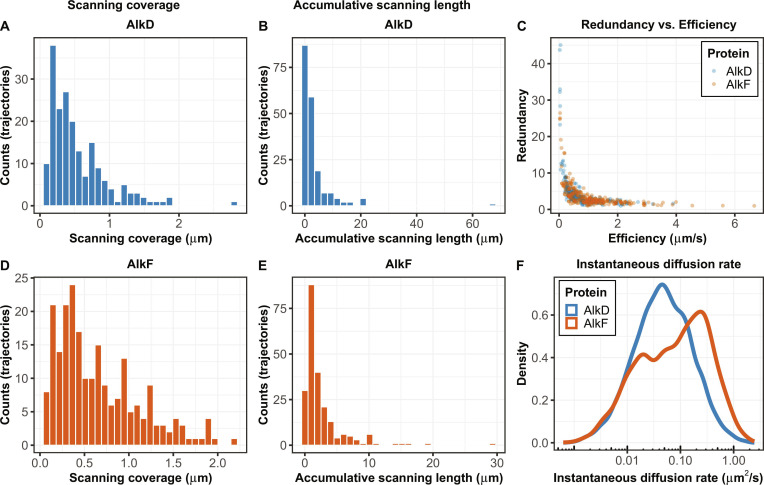
Quantitative descriptors of substrate scanning behavior. (A and D) Distributions of scanning coverage, defined as the maximal substrate length covered by a protein during a single trajectory, shown for 2 exemplary proteins (AlkD and AlkF). (B and E) Distributions of accumulative scanning length, calculated as the cumulative sum of absolute frame-to-frame displacements for each trajectory. (C) Color-coded redundancy–efficiency analysis, where redundancy is defined as cumulative scanning length divided by scanning coverage, and efficiency as scanning coverage divided by trajectory duration. (F) Distribution of instantaneous diffusion rates calculated using a rolling window mean squared displacement approach, revealing multimodal scanning behavior that is not captured by trajectory-averaged diffusion coefficients.

Scanning coverage for each trajectory is calculated as the maximal stretch of the substrate that is covered by the protein in that trajectory. Scanning coverage can be interpreted as a measure of the glide of the protein on the substrate. Smaller values indicate confinement of the movement to shorter stretches, while larger numbers suggest greater freedom of movement along the substrate. The average scanning coverage is determined by averaging the scanning coverage values for all trajectories. The distribution of scanning coverage of trajectories for the 2 exemplary proteins (AlkD and AlkF) are shown in Fig. 4A and D.

In thermally driven movements, proteins constantly change the direction of the scanning. Therefore, the total distance they travel along the substrate is usually larger than their net start-to-end displacement. Accumulative scanning length is calculated as the cumulative sum of the absolute values of frame-to-frame displacements for each trajectory. This can be interpreted as a measure of how rigorously the substrate is scanned, which is determined by how fast the proteins can move and how frequently they change the direction of scanning. For example, in the case of thermally driven scanning of DNA, a larger number for the accumulative scanning length means longer travelled distance along the substrate per trajectory. In the case of ATP-driven unidirectional translocations, the accumulative scanning length represents the start-to-end displacement, which is equal to scanning coverage. Figure 4B and E shows distributions of accumulative scanning lengths, respectively, for 2 different proteins.

### Redundancy–efficiency and diffusion rate

Redundancy–efficiency analysis is particularly relevant for thermally driven scanning of substrates, where the scanning involves target localization. Redundancy is defined as the average number of times any given point along the substrate can be visited within the scanning range of each trajectory. Redundancy for each trajectory is calculated by dividing the accumulative scanning length by the scanning coverage length. Efficiency is defined as the rate at which a maximal length of substrate is covered in each binding and scanning event by the protein. The value is calculated as the scanning coverage divided by the trajectory duration. Proteins with high redundancy tend to scan within confined regions of the substrate, potentially looking for targets in short and local stretches. In contrast, proteins with high efficiency can cover longer stretches of the substrate, potentially searching for targets positioned remotely along the substrate. Figure 4C shows color-coded distribution of redundancy–efficiency data for different proteins.

Diffusion rate analysis is a commonly used approach for characterizing random movements. One caveat of the approach is that the diffusion rate is usually calculated as an average value over the entire length of the trajectories. Hence, variations of the diffusive movements within trajectories are not detectable in these calculations, as they will be averaged out in the traditional form of calculation of the diffusion rate. To address this, we use a rolling window function to calculate the instantaneous diffusion rate from the mean squared displacement of the movement within this window. This calculation is repeated over all frame-to-frame displacements of the trajectories, providing values of instantaneous diffusion associated with each particular frame. Hence, the distribution of instantaneous diffusion rate values is a much more informative analytical approach compared to the average values. For example, as shown in Fig. 4F, the multimodality of scanning [1,3] will be indicated by existence of more than one peak in the distribution plot, which would not be detected by only looking at the average value of the diffusion rate for that protein.

## Discussion

### Applications of the method

The SMITracker platform can be used for efficient analysis of large datasets from single-molecule experiments involving molecular scanning or translocation along substrates with linear structures such as DNA [1,10], microtubule [17], or actin filaments [18] that are elongated in a linear form. A typical dataset from such experiments includes a sequence of recorded images, in which the movement of fluorescently labeled proteins (or protein complexes) along the linear track of the substrate can be traced. These movements can be either thermally driven or performed by various molecular motors embedded in the structure of the proteins. Depending on the type of data, SMITracker can be applied in several ways. The application of SMITracker can be divided into 4 stages with intermediate output files from each stage, providing flexibility for partial use of the platform. These 4 stages include (a) data transformation, where users transform imaging data to structured data for subsequent steps or continue with an independent approach for the rest of the analysis; (b) trajectory detection, where SMITracker performs initial spatial filtering of the data and subsequent trajectory identification; (c) noise exclusion, where SMITracker produces a tidy set of interaction data ready for further diffusion analysis (the intermediate data produced in this stage also enable researchers to continue with their preferred analysis using other tools and approaches if so wished); and (d) diffusion analysis, where SMITracker is used to its full potential, providing deep mechanistic insight of the scanning process.

The output of the last diffusion analysis step includes scanning speed, trajectory duration, scanning coverage, scanning accumulative length, redundancy–efficiency of scanning, and instantaneous diffusion rate analysis. Users can obtain the average value for these parameters and investigate their distributions in detail. The platform allows for the analysis of an unlimited number of proteins enabling comparisons between datasets in the context of the aforementioned parameters. Additionally, up to 5 different experimental conditions (in the form of metadata) can be associated with each dataset, allowing for quantitative investigation of these conditions’ effects on the various parameters.

### Comparison with other methods

There have been a number of interesting open-source tracking platforms developed recently [30–34]. Some of these platforms have been designed for tracking cells and efficiently detecting cell migration, differentiation, and fusions [33,34]. Although they are effective for analyzing data from specific experiments for which they were developed, they encounter practical limitations when analyzing large single-molecule scanning datasets collected at high image frequency. Target data for these platforms are sequences of frames collected at low frequency and with a limited number of events to track. Utilizing these platforms for analyzing single-molecule DNA scanning data collected at very high frequencies, where thousands of short-lived interactions might occur in one dataset, can require substantial parameter tuning and manual intervention during trajectory detection. In studies of single-molecule scanning, comparative analysis across different proteins and experimental conditions is often central. To obtain valid results from such comparisons, it is important that datasets are processed in a consistent manner and with minimal user-introduced bias. SMITracker addresses this by automating the trajectory detection step and implementing an adaptive noise-exclusion framework in which classification parameters are recalculated automatically for each dataset. While users may adjust the overall stringency of the filters, these settings are applied uniformly across the entire datasets, helping to promote minimal user intervention, reproducibility, and consistent treatment across experiments.

To reduce the computational cost of dealing with large datasets, SMITracker separates signal localization from downstream tracking and analysis. Fiji-based processing is restricted to signal localization using ThunderSTORM, which is optimized for handling large image stacks typical of super-resolution microscopy. Localized signals are imported into R, where they are consolidated into matrix-based representations that facilitate efficient trajectory reconstruction and noise-exclusion analysis. By operating primarily on localization coordinates rather than full image stacks during downstream processing, SMITracker reduces repeated loading and manipulation of large image files, which can become limiting for image-based workflows when applied to large and noisy single-molecule datasets. Under typical conditions, the most computationally demanding steps—signal localization and core tracking analysis—require on the order of minutes for datasets of approximately 100,000 frames, offering performance that is well suited to the scale and demands of single-molecule scanning experiments.

## Conclusion

Here, we have introduced the SMITracker platform, designed to address common challenges in single-molecule experiments involving linear substrate scanning. This multi-module platform assists with data organization and transformation, trajectory detection, noise exclusion, and visualization of results. The automatic trajectory detection algorithm saves considerable time and allows for the analysis of extremely large datasets, extracting large amount of high-quality data, while uniform noise exclusion minimizes user bias. Moreover, the multifaceted visualization tools provide deep insights into the mechanistic features of scanning dynamics. SMITracker is delivered through an R Shiny interface, available as an R package for R users and as a Docker container for non-R users. This platform enhances productivity and data quality in the analysis of single-molecule substrate scanning experiments.
