# Supplementary material for: SMITracker: An Interactive Platform for Tracking and Analysis of Single-Molecule Interactions with Linear Substrates
Source: Comput Struct Biotechnol J. 2026 Mar 18;35(1):0014. doi: 10.34133/csbj.0014 (PMC13047747; doi:10.34133/csbj.0014)
Supplement: Supplementary 1 — Supplementary Text Figs. S1 to S7 [file csbj.0014.f1.pdf]

SMITracker: an interactive platform for tracking and analysis of single-molecule interactions with linear substrates Supplementary Material

Arash Ahmadi[1,*], Magnar Bjørås[2,3], Bjørn Dalhus[3,4,*]

1. Centre for Computational and Data Science (dScience), Faculty of Mathematics and Natural Sciences, University of Oslo, PO Box 1066 Blindern, N-0316 **Oslo**, Norway
2. Department of Clinical and Molecular Medicine, Faculty of Medicine and Health Sciences, Norwegian University of Science and Technology (NTNU), PO Box 8905, N-7491 Trondheim, Norway
3. Department of Microbiology, Oslo University Hospital HF, Rikshospitalet and University of Oslo, PO Box 4950 Nydalen, N-0424 Oslo, Norway.
4. Department of Medical **Biochemistry**, Institute for Clinical Medicine, University of Oslo, PO Box 4950 Nydalen, N-0424 Oslo, Norway

* Corresponding authors: arash.ahmadi@dscience.uio.no and bjorn.dalhus@medisin.uio.no

Preparation

Sample dataset

The sample datasets provided in this GitHub repository (https://github.com/ArashAh/SMITracker_sample_data) are a small subset of a much larger collection processed in our previous work. These JSON files are the output of the preprocessing of raw image data that is done using ThunderSTORM plugin in Fiji. These datasets are intended as examples to test the platform's operation, not for drawing conclusions about the corresponding proteins, as the detailed results have already been published. (Ahmadi et al. Commun. Biol. 4, 2021).

In addition to preprocessed JSON files, the repository includes set of intermediate output files produced by each modules of SMITracker. We have also provided a sequence of interface screenshots during the creation of these intermediate files. These can serve as a visual guide to complement the detail explanations in the Procedure section. Depending on the user input across different modules, the content of newly produced intermediate files might slightly differ from those we have provided. Therefore, a row-by-row comparison of outcomes may vary due to user-applied spatial filtering not be exactly replicated. However, as long as the user defines the substrate's approximate location, any deviations will be minor, primarily affecting non-target data and labelling and arrangement of the target data. Importantly, the final target data and the outcomes of the last module are significantly replicable.

Procedure

The procedure is divided into 7 separate steps and each step contains sub-steps that are identified with a number, defining the respective operation in that sub-step. Moreover, each sub-step might have comments or explanations to address important points about the operation in that step, separated by alphabetic letters.

1. Preprocessing of raw data

1. Use the following naming convention for the TIFF files to provide the metadata with each dataset: *ProteinName_FrameInterval_ExperimentalCondition1_ExperimentalCondition2_ExperimentalCondition3_ExperimentalCondition4_ExperimentalCondition5_UniqueIdentifier_ non-metadata.tif*
    a. Protein name and frame interval (in milliseconds) are the minimum essential information that should be provided with every dataset.
    b. The order of the elements in the file name does not matter and their positions can be switched as long as they can be addressed accordingly in step 2.4.
    c. Experimental conditions can be a numerical value corresponding to e.g. salt concentration, pH, etc., or they can represent categorical variables as a character string with information such as presence of particular chemical agents.
    d. If the experiment does not involve any dataset-specific experimental conditions, they can be left out of the dataset names.
    e. The file names should be unique to each dataset; if all the parametric name parts of two or more datasets are the same, then a unique identifier field at the end should be added to generate unique file names for the two identical experiments.
    f. Any part of the file name that does not contain any relevant metadata (non-metadata), such as repetitive suffixes generated by the recording software, can be placed at the end of filenames before filename extension.
    g. Example file name: AlkD_33.5mspf_10mMKCl_20240717_15000x_pH7_1_MMStack_Pos0.ome.tif
    This represents a dataset for protein AlkD collected at a frame rate of 33.5 millisecond per frame, in a 10 mM salt concentration, on 2024-07-17, with a 15000X dilution of the original batch at pH 7, and a unique identifier set as 1. The remaining components are considered non-metadata parts of the filename.
2. Copy all the TIFF files into a folder named *TIFF_ProteinName.*
    a. As a common best practice in data management, it is recommended to keep an original version of the raw data as backup, as there is a chance that the data would be modified in the next step.
    b. The naming of the folder is arbitrary, but the folder should only include the files intended for the analysis.
3. Open datasets one-by-one in Fiji and use "*image>stacks>Plot Z_axis Profile*" to check for potential frames with saturated signals and remove these frames from the datasets and save the changes.
    a. If a dataset contains saturated frame(s), the corresponding JSON file will be corrupt and cannot be loaded into R. In addition, instead of one single projection file corresponding to each TIFF file in step 1.9, the program produces an image stack extracted from the original TIFF.
4. Create two other folders next to the folder in step 1.2, and name them as *JSON_ProteinName* and *Projection_ProteinName.*
5. In Fiji, use the "*Plugins>thunderSTORM>Camera setup*" menu to adjust the camera setup of the plugin to match the camera parameters used in the experimental setup.
    a. The camera setup used for signal localization of the provided sample datasets are: *Pixel size [nm]: 112, Photoelectron per A/D count: 20.02, Base level [A/D counts]: 991.34, EM gain: 398.01*

   b. The user needs to retrieve these numbers based on camera specification and the optical setup of use.
6. Follow the next steps (1.7-1.8) for automatic signal localization of the entire data or perform the localization analysis file by file using the *thunderSTORM* user interface. Save the output JSON and projection files into folders `JSON_ProteinName` and `Projection_ProteinName`, respectively, and jump to step 1.9.
7. Inside FIJI, open the file provided in this GitHub link: https://github.com/ArashAh/SMITracker/blob/main/localization_analysis.ijm and run the script.
8. After running the script, the program will ask the user for the location of folders three times; direct it to the folders made in step 1.2 and 1.4, in the following order: `TIFF_ProteinName`, `JSON_ProteinName` and then `Projection_ProteinName`.
   a. The program will now automatically perform single-molecule localization on all datasets in the `TIFF_ProteinName` folder, and for each dataset it will produce a JSON file (saved in folder `JSON_ProteinName`) along with a TIFF file (saved in folder `Projection_ProteinName`) showing the projection of all signals detected in each dataset into one image. The filenames provided in the initial TIFF files will be preserved and used to name the corresponding JSON and TIFF output files.
   b. Depending on the number of observations in each dataset, the localization process might take 1-15 minutes for each dataset.
9. Inspect the produced TIFF projection files and see if the trace of fluorescence signals can be identified where the substrate is supposed to be.
   a. Examples of these projection figures are presented in Fig.1, where traces of proteins scanning along the substrate are readily visible.
   b. This first visual assessment can give a rough overview of the quality of the data. The more discernible this accumulation of signals around substrate are compared to the rest of the field of view, the higher amount of target data can be acquired from that dataset.
   c. The positions of the localized signals are reported with the unit of nanometres in *ThunderSTORM*, which is also used for reporting distances in SMITracker unless otherwise stated.
10. Before continuing with further steps of the analysis in SMITracker, the user needs to calculate an approximate localization precision of the microscope for the same fluorescent dye with a similar range of laser power, exposure time and camera gain.
    a. A few sets of signals from dyes attached to the surface of the coverslip (stationary signals) is needed to calculate the localization precision. Labelled proteins randomly associated to the surface of the coverslip in any dataset can be used for this purpose.
    b. If no such particle can be found, the user can immobilize labelled proteins on a surface of an untreated coverslip and record a few hundreds of frames for a few particles.
    c. Perform manual signal localization using *ThunderSTORM* user interface on the abovementioned stationary signals and extract x- and y- coordinates of those signals in separate columns.
    d. Calculate the standard deviations (e.g. use *sd()* function in R) of the emitted signal in the x- and y-direction for a few fixed particles (minimum 3) and calculate their average for each direction separately (x and y); these numbers will be used in step 4.5.

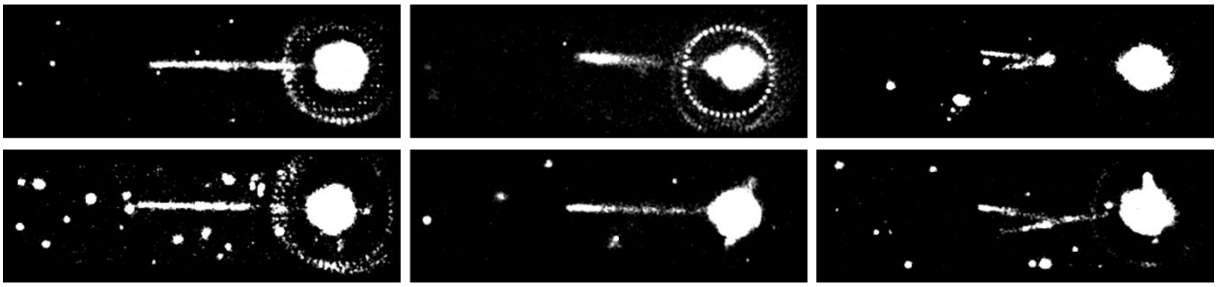

Fig.1 | Projection of localized signals. Exemplary outputs of the localization process where all signals in each dataset are localized and projected onto single images. The bright bulk of signals on the right side of the figures are the result of accumulation of proteins on the polystyrene beads. The linear accumulation of signals protruding from the beads are traces of labelled proteins scanning DNA. The two panels on the right show examples where two DNA molecules are attached to the same bead.

2. Loading data into SMITracker

To install and run SMITracker as an R package or Docker container, follow the instructions in the GitHub repository (https://github.com/ArashAh/SMITracker/releases/tag/v1.0.0). All analyses were performed using SMITracker v1.0.0, and the following instructions apply to this version. Upon running the platform inside R or a container, the interactive interface will be available over a *localhost* on the browser. Fig.2 shows the first module of SMITracker, and the numbers in the procedure refer to the distinct parts of the interface as shown.

1. Navigate to the folder where JSON files are stored and select files to upload.
2. Check the format of the file names here, and ensure they follow a uniform naming convention (as explained in step 1.1). Copy the unique part of the file name containing the metadata, and exclude non-metadata parts e.g. "`MMStack_Pos0.ome.tif.json`" in the case of the sample datasets.
3. Paste the copied text from the previous step into this box.
    a. This text string carries the metadata associated with the dataset, which will be defined in the next step.
4. This array of dropdown menu represents different parts of the filename. Using the menu for each part, specify what metadata that part of the file name represents:
    a. Protein name and time interval are required information that must be provided with file names for all datasets.
    b. Time interval should be given in milliseconds.
    c. Up to 5 different experimental conditions can be associated with each file name.
    d. If there are fewer than 5 experimental conditions, leave the rest as NA.
    e. If any part of the selected file name does not carry essential information about the experimental conditions, leave that position as NA.
5. Type the separator of the distinct parts of the file name; the default is "_".
6. Select the experimental conditions that represent numerical values.
7. Type a unique analysis identifier; all the intermediate and the final output files will be saved with this identifier as a part of their file name.
8. Load and convert the JSON files. Depending on the number of observations per dataset, loading and transforming of data might take between 3 – 60 seconds for each JSON file.
9. After the process is completed, a summary of the imported data, including the number of frames and observations for each dataset, will be shown in this summary table.
10. Here, a detailed list of datasets with number of frames and observations are provided.
11. Save the result of the process by pressing the *Download Processed Output* button. The file will be saved with the following naming convention:

`1_data_loading_analysisIdentifier_date.rds`; this file will be the input for the next module.

12. Save a visual log of the module; this will download a snapshot of the analysis in its current form and will be useful for later references.

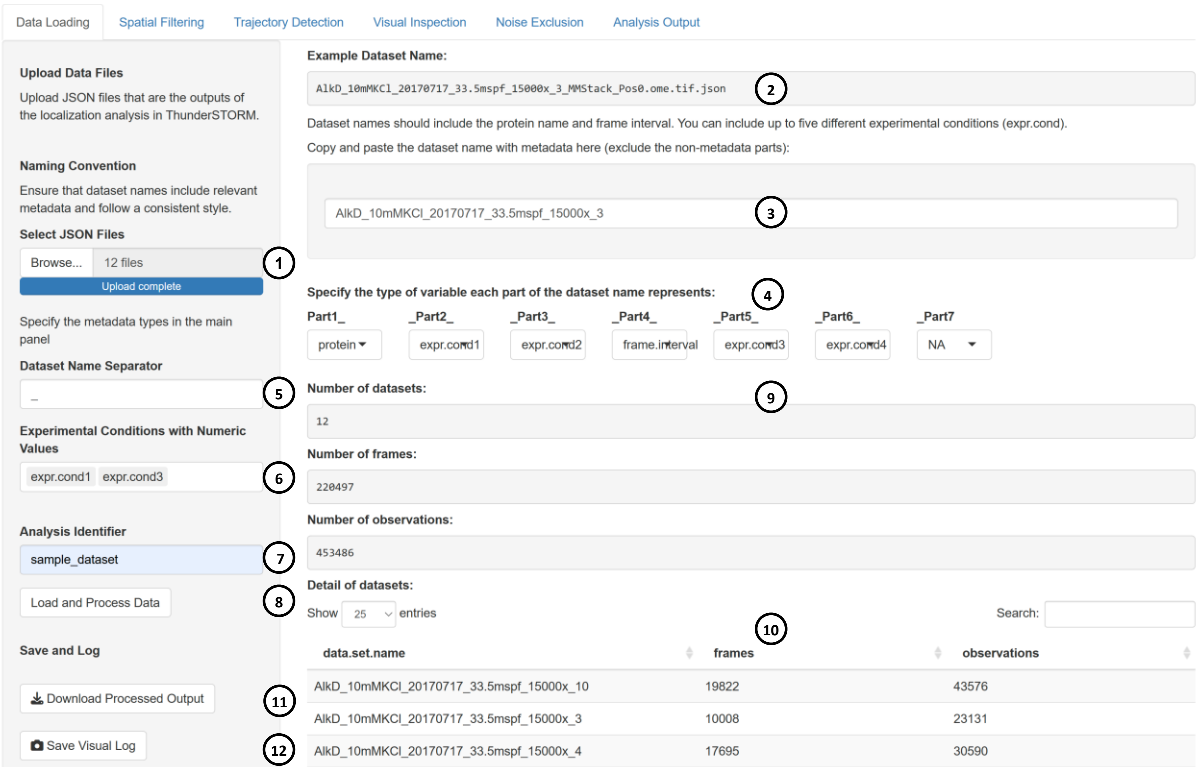

Fig.2 | First module of the SMITracker platform (Data Loading). Preprocessed JSON files are imported and transformed into structured data-fame format. The user can get an initial overview of the imported data, including the number of frames and observations per dataset here. Each part of the figure has been marked with numbers corresponding to the steps in the procedure with detailed explanation in the text.

3. Spatial filtering

Fig.3 shows the *Spatial Filtering* module of SMITracker, and the numbers in the procedure refer to different parts of the interface as shown.

1. Navigate to the file saved from the *Data Loading* module and press the *Start Processing* button.
2. The name of the current dataset will be shown in this box.
3. Navigate through different datasets using this numeric input. Follow through step 5-10 for each dataset at the time.
4. Here the initial result of the signal localization is visualized. For each dataset, select a rectangular region of interest (ROI) and make sure to include all potential traces of the proteins scanning the substrate.
    a. The ROI does not need to be drawn very tightly around the trace of the molecules moving on the substrate. The purpose is to exclude large parts of the field of view that do not carry the target data. The automatic and accurate detection of trajectories of proteins scanning substrate takes place in the next module.
5. After defining a ROI for each dataset press the *Substrate Trace* button.
6. The selected ROI for the current dataset can be seen in this plot.
    a. The selected ROIs for each dataset are saved upon selection and the user can inspect them by navigating between the datasets.

7. For each dataset select a few ROIs where signal comes from a surface-bound molecule and press the *Surface-bound Signal* button.
8. In datasets with two substrates in one field of view, follow steps 4-6 once for the first substrate and then repeat the process for the next elongated substrate, only with the difference that after selecting the ROI for the second substrate (in step 5), this time press the *Split Substrate Trace* button instead of the *Substrate Trace*.
9. Keep track of the datasets being spatially filtered using the list shown here.
10. After selecting the ROIs (substrate trace and surface-bound signals) move to the next dataset by pressing the *Next Dataset* button
11. If needed, press the *Reset Current Selection* button to reset the selected ROI for the current dataset and repeat the process of ROI selection for that dataset.
12. After going through all datasets press the *Show Intensity Distribution* button to see the distribution of intensity of the localized signals.
13. See the distribution of signal intensity in this plot.
    a. By looking at the limits of intensity distribution for the substrate trace and Surface bound signal, the user can define an approximate range for acceptable intensities.
    b. The purpose is to exclude extreme values of intensity, later, in the noise exclusion module, a more accurate intensity filter will be applied.
14. Define the maximum and minimum intensity filter to exclude extremely high or low intensity noise from the data.
    a. In this step, the user can use the plot shown in the previous step to define the approximate limits of intensity.
    b. The purpose is to exclude the signals with extreme values of intensity from the target data. However, in the case of very accurate photon counting detection systems, the user might choose to set this limit tight according to the accuracy with which they can define the number of photons received per signal per exposure time.
    c. Care must be taken here; the signal from single fluorophores or Qdots received by a normal EMCCD camera can be highly variable, setting a tight limit in this step might lead to exclusion of parts of data as being noise.
    d. Later, in step 6.4 a more accurate intensity filtering system will be applied in the noise exclusion model.
15. Save the result of the processing by pressing the *Download Processed Output* button. The data will be saved with the following naming convention `2_spatial_filtering_ analysisIdentifier_date.rds`
16. Save a visual log of the module in its current state.

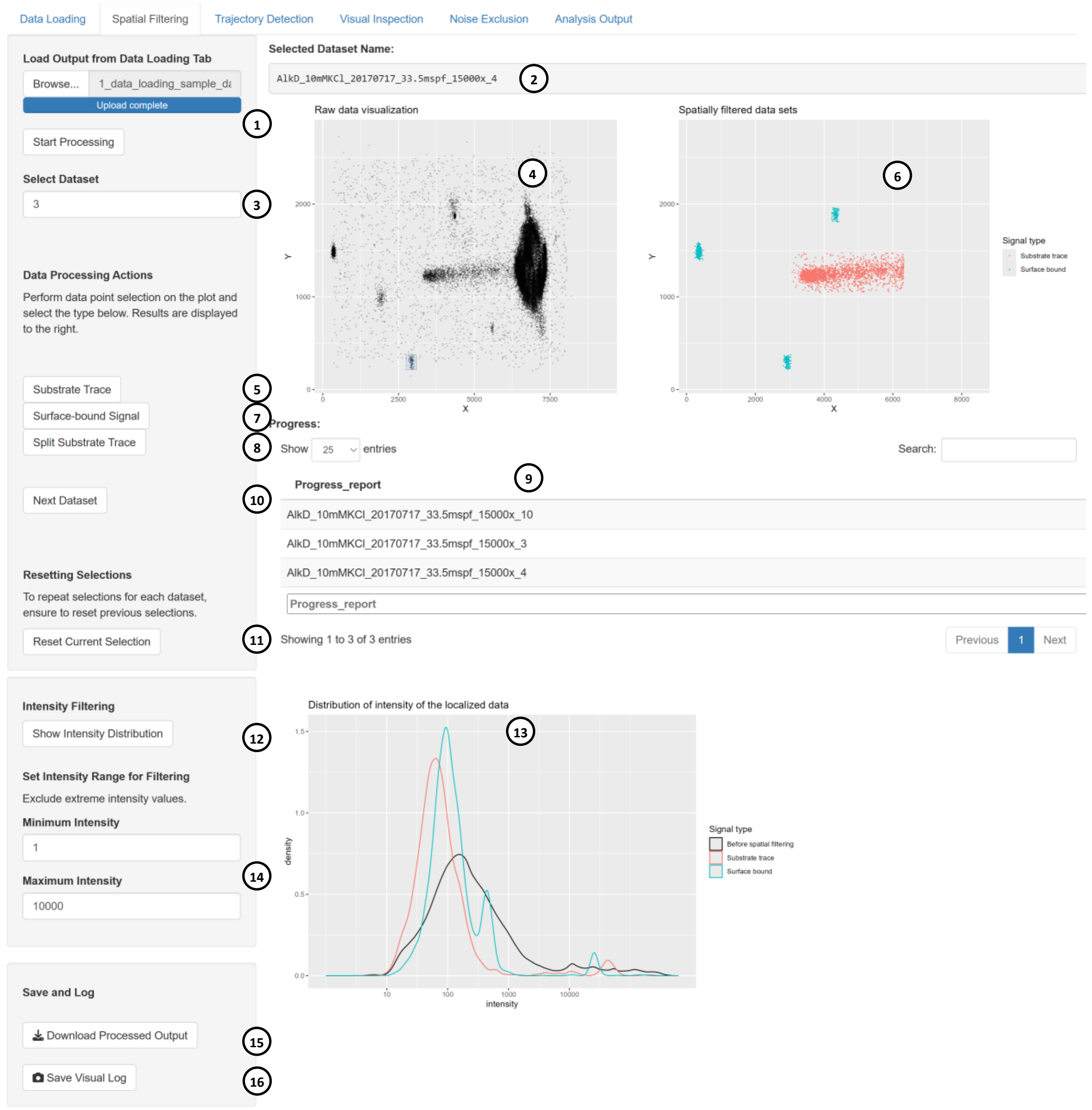

Fig.3 | Second module of the SMITracker platform (spatial filtering). Datasets imported from the previous module are visualized individually in the spatial filtering module, and the user can define the approximate position of the substrate, as well as a few surface-associated signals, to be used for the noise exclusion model. Each part of the figure has been marked with numbers corresponding to the steps in the procedure with detailed explanation in the text.

4. Trajectory detection

Fig.4 shows the *Trajectory Detection* module of SMITracker and the numbers in the procedure refer to different parts of the interface as shown.

1. Navigate to the file saved from the *Spatial Filtering* module and upload the file.
2. Give an approximate maximum value for frame-to-frame displacement (in nanometers) of proteins along the substrate.
    a. Numbers entered in steps 4.2 and 4.3 are initial approximations for the tracking algorithm.

b. The default number shown in the interface is a good starting point for most of the thermally-driven single-molecule scanning data. Otherwise, follow the steps below to calculate these values for specific datasets.
c. Find a few trajectories in the imaging platform (in this case Fiji)
d. Measure the frame-to-frame displacement of the protein along the substrate in the x-direction (in nanometers) and repeat this for 10 different frame intervals.
e. Calculate the average (*mean*) and standard deviation (*sd*) for these 10 measurements.
f. Type in the outcome of the *mean* + *3* * *sd* as an approximation of the maximum frame-to-frame displacements.

3. Repeat the previous step and insert the frame-to-frame displacement for displacement across substrate (y-direction).
4. Insert the average localization precisions in the x- and y-directions from the numbers calculated in step 1.10 in this box.
5. Define the lower threshold for the number of frames per trajectory; this number depends on the binding lifetime of the protein as well as the image capture frequency. In the case of very short-lived trajectories, we recommend at least 5 frames as a limit here, but for data where interactions occur over rather longer periods of time or data with much noise the user can increase this number.
6. Define how many trajectories per dataset should be used for optimizing the parameters of the noise exclusion model.
    a. The best trajectories for optimizing the noise model are those with the longest range of movement along the substrate.
    b. The larger the number of trajectories per dataset used for optimizing the parameters of the noise exclusion model, the higher the accuracy of the model. However, there is a trade-off here; the user needs to visually inspect those trajectories in the next module. Therefore, selecting too many trajectories here can make the next module time consuming. We recommend between 5 and 10 trajectories per dataset.
    c. Care must be taken as datasets with lower total number of trajectories than the value defined here, are excluded from further analysis. Therefore, in cases where the total number of trajectories per dataset is typically very low (e.g. below 10), it is better to go with a smaller number here (closer to 5).
7. After all the initial values are given, press the *Detect trajectories* button.
    a. Depending on the number of observations in each dataset, the processing can take 30 seconds to 10 minutes per dataset.
8. Once the tracking is complete, plots showing the distribution of step sizes of trajectories in the x- and y-directions are produced.
    a. These plots show the frame-to-frame displacement distributions.
9. This plot shows the proportion of frames that are going to be used for optimizing the noise exclusion model parameters (cyan bars) to the total amount of data. This plot can be used to make an informed guess about the proportion. Based on this the user can decide to tweak the number of selected trajectories and re-analyze, if needed. The figure is not shown until the tracking has been completed at least once.
10. If the maximum frame-to-frame displacement numbers (in steps 4.2 and 4.3) are grossly underestimated, the algorithm will return a warning here, suggesting that the user increase those numbers by a certain amount and repeat the analysis.
    a. This message will not be shown until all steps have been performed and the tracking analysis has been completed at least once.

11. If the maximum frame-to-frame displacement value is not accepted as described in the previous step, increase the displacement by the stated amount in in the warning issued in the previous step and press the *Re-analyze with New Parameters* button.
12. Press the *Visualize Detected Trajectories* button to visualize the result of the tracking for a specific dataset.
13. Navigate through datasets by entering the dataset number.
14. The results of the tracking analysis can be inspected here
    a. The red and cyan data points represent detected trajectories and surface bound signals, respectively.
15. By pressing the *Download Processed Output* button, the data will be saved with the following naming convention: `3_trajectory_detection_analysisIdentifier_date.rds`

16. Save a visual log of the module in its current form.

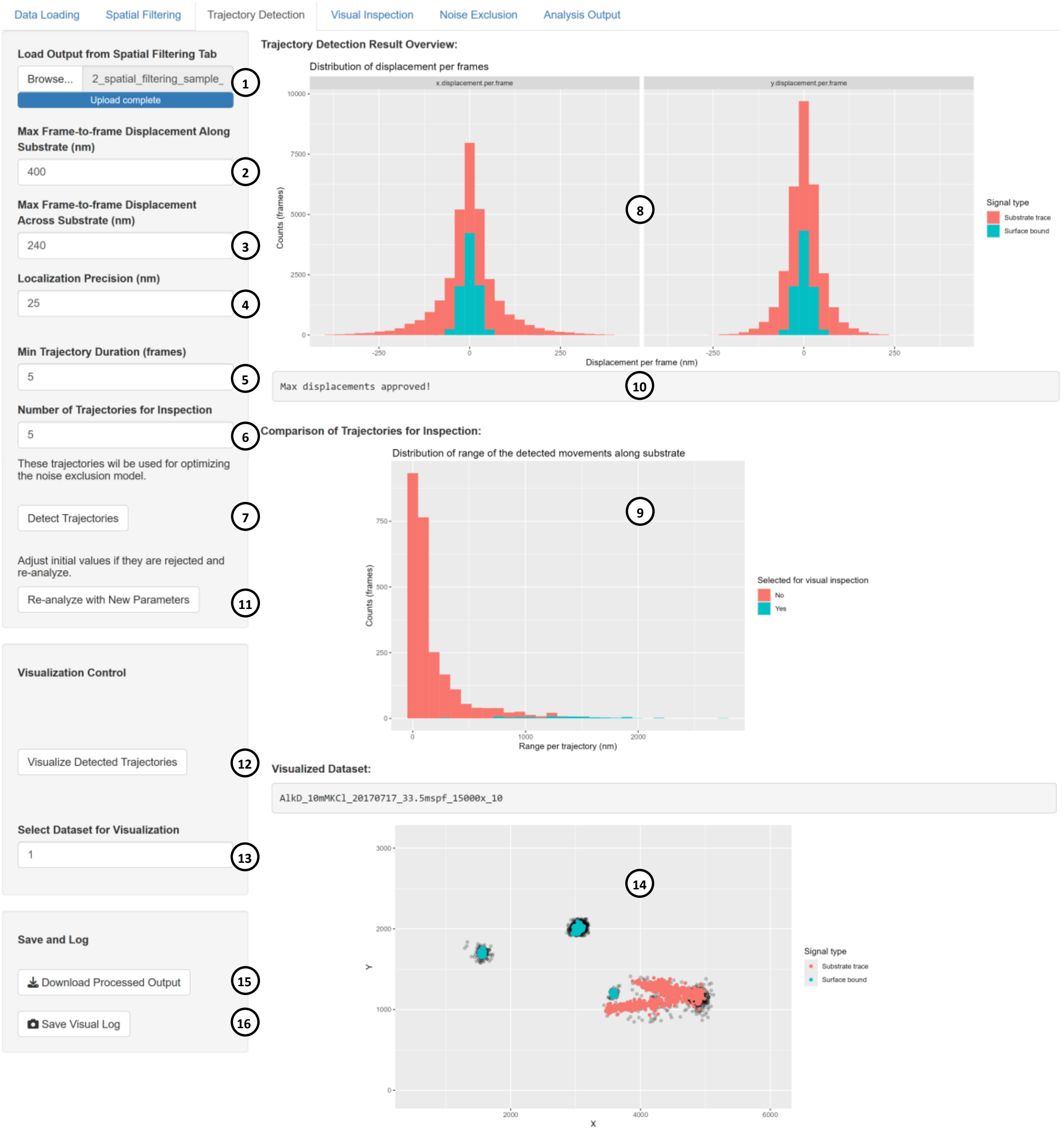

Fig.4 | Third module of the SMITracker platform (trajectory detection). In this module, the automatic trajectory detection algorithm runs on the entire datasets A few initial values are needed for the algorithm to begin, with clear instructions for how to extract those numbers by inspecting a small subset of the data. Each part of the figure has been marked with numbers corresponding to the steps in the procedure with detailed explanation in the text.

5. Visual inspection

Fig.5 shows the *Visual Inspection* module of SMITracker and the numbers in the procedure refer to different parts of the interface as shown.

1. Navigate to the file saved from *Trajectory Detection* module and press the *Visualize Trajectories* button.
2. Navigate through the different datasets.
3. See the name of the current dataset here.
4. Navigate through the different trajectories inside the current dataset.
5. See the trajectory-specific ID here.
6. See the x-y coordinates of the collection of representative trajectories in the current dataset projected onto a single image.
    a. The collection of all trajectories is colored red, with the current trajectory in black.
7. See the x-displacement time series for the current trajectory.
    a. Inspect if the movement looks like as expect (a normal random walk in case of thermally driven movement or a direct movement in the case of ATP-driven translocation) and it is not an artifact.
    b. In case the movement seems anomalous compared to the rest of the trajectories, that trajectory should be removed (in step 5.8) from the dataset that is going to be used to optimize the parameters of the noise exclusion model. Inspect the corresponding range of frames in the original TIFF file, if necessary.
    c. Keep an eye on the intensity alarm. The color of the datapoint in this plot turns into red if the intensity (number of photons) of the localized signal is categorized as outliers compared to the rest of the data. There could be two scenarios in these cases; either a large object has collected many fluorescent dyes and has passed the field of view, or an aggregate of proteins is interacting with the substrate. These cases can be identified by checking the original TIFF files, and such trajectories should be removed from the optimization dataset.
    d. Given the dataset name in step 5.3 and the frame number shown in the plot in this step, the user can localize the corresponding trajectories inside the TIFF file.
8. Press the *Remove Trajectory* button to remove the current trajectory from the optimization dataset if it is not a representative trajectory suitable for optimization of the noise exclusion model.
    a. Cycle through all trajectories in all datasets and discard any poor trajectory according to step 7.
9. Save the visually inspected data by pressing the *Download Processed Output* button. The following naming convention is used: `4_visual_inspection_analysisIdentifier_date.rds`
10. Save a visual log of the module in its current form.

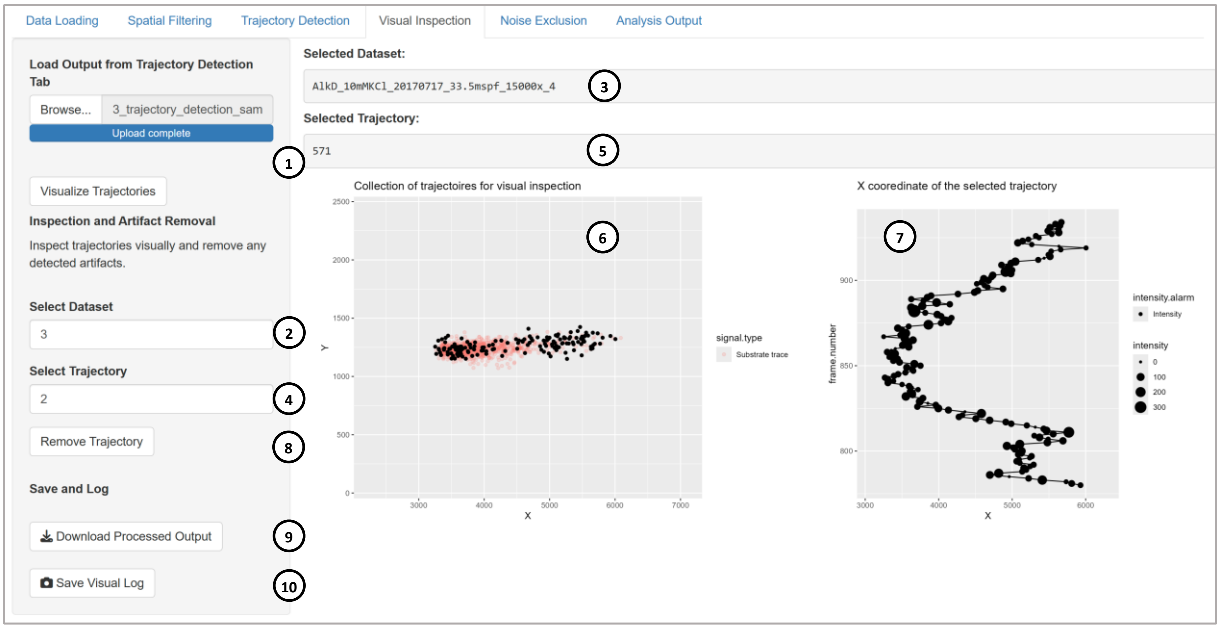

Fig. 5 | Fourth module of the SMITracker platform (visual inspection). In this module, the user needs to inspect a number of trajectories for each dataset. The trajectories should represent the normal scanning of substrate by labelled proteins. These trajectories are going to be used for optimizing the parameters of the noise exclusion model in the next module. Each part of the figure has been marked with numbers corresponding to the steps in the procedure with detailed explanation in the text.

6. Noise exclusion

Fig.6 shows the *Noise Exclusion* module of SMITracker and the numbers in the procedure refer to different parts of the interface as shown.

1. Load the data saved from the *Data Loading* module.
2. Load the data saved from the *Spatial Filtering* module.
3. To perform the noise exclusion, press the *Apply Noise Filters* button.
    a. The settings discussed in 6.4 and 6.5 can be tweaked after this step as well, and the user can see the effect of the changes on the visualizations immediately.
4. Select the type of noise to include in the initial visualization (shown in step 6.7).
    a. Different sources of noise are color-coded in the plot shown in step 6.7.
    b. By deselecting any of the items in this list the corresponding noise will be removed from the visualized data.
    c. The noise selecting and deselecting function in this module is only for the purpose of visualization and does not affect the function of the noise exclusion model.
5. Define how strictly (smaller values) and how loosely (larger values) these different types of these filters are going to be applied.
    a. The values defined here are applied to all datasets uniformly and changes are applied to both the visualizations as well as to the final selection of the noise excluded data.
    b. The default numbers for these gauges produce reasonable parameters in most cases but based on the general level of the signal-to-noise ratio, the user can tweak these values.
    c. Too strict filters can lead to erosion of data by removal of true signals in datasets with high fluctuations in the measured parameters. Vice-versa, too loose filters might lead to inclusion of noise signals as data points.

    d. The user should try to find filter values where the model performs reasonably on most of the datasets. However importantly, to avoid introducing any biases the filters are applied homogenously on all datasets.
6. Navigate through different datasets selecting the respective number.
7. The figure shows all detected signals from each dataset with original x-y coordinates.
    a. The grey data points show data that has been labelled as noise and excluded from the rest of the analysis due to effect of operations in module 2 and 3, before applying the noise exclusion model.
    b. The color-coded data points show results after applying the noise exclusion algorithm, with red points being the final data passed on to the diffusion analysis. Excluded data points are color-coded according to different categories as listed next to the plot.
    c. Notice that if a particular type of noise is not present in the current dataset, that category does not show in the plot legend even if the corresponding signal type is selected in step 6.4
8. This figure shows collection of (only) noise-excluded data in a rotated coordinate system with the trace of the scanning molecules projected horizontally in the x-direction.
    a. Red data points show the total collection of all noise-excluded trajectories in the current dataset, while the black data points show data for any particular trajectory selected in the next step.
9. Press the *Visualize Trajectories* button to visualize trajectories one-by-one in the x-y coordinate system (black data points in step 6.8) as well as time series of x and y displacements separately in steps 6.11 and 6.12
    a. The data points in the trajectories visualized in this step are synchronized with the items selected in step 6.3 and filters applied in step 6.4.
10. Navigate through the different trajectories for each dataset.
11. This plot shows the displacement of the proteins along substrate in time.
12. This plot shows the displacement of the proteins across substrate in time.
    a. From the last two plots, the user can investigate trajectories in detail one-by-one.
    b. The user can perform such detailed investigation on a randomly selected set of trajectories to assess how the model is performing.
    c. If needed, the user can investigate the original TIFF dataset by navigating to the range of frame numbers shown in these figures and observe the original signals related to that particular trajectory.
    d. The user cannot remove or add any data point in any of the data categories manually. The contents of the data in those categories are solely defined by the filters applied in step 6.4.
13. Press the *Download Processed Output* button to save the data with the following naming convention: `5_noise_exclusion_ analysisIdentifier_date.rds`
14. Save a visual log of the module in its current form.

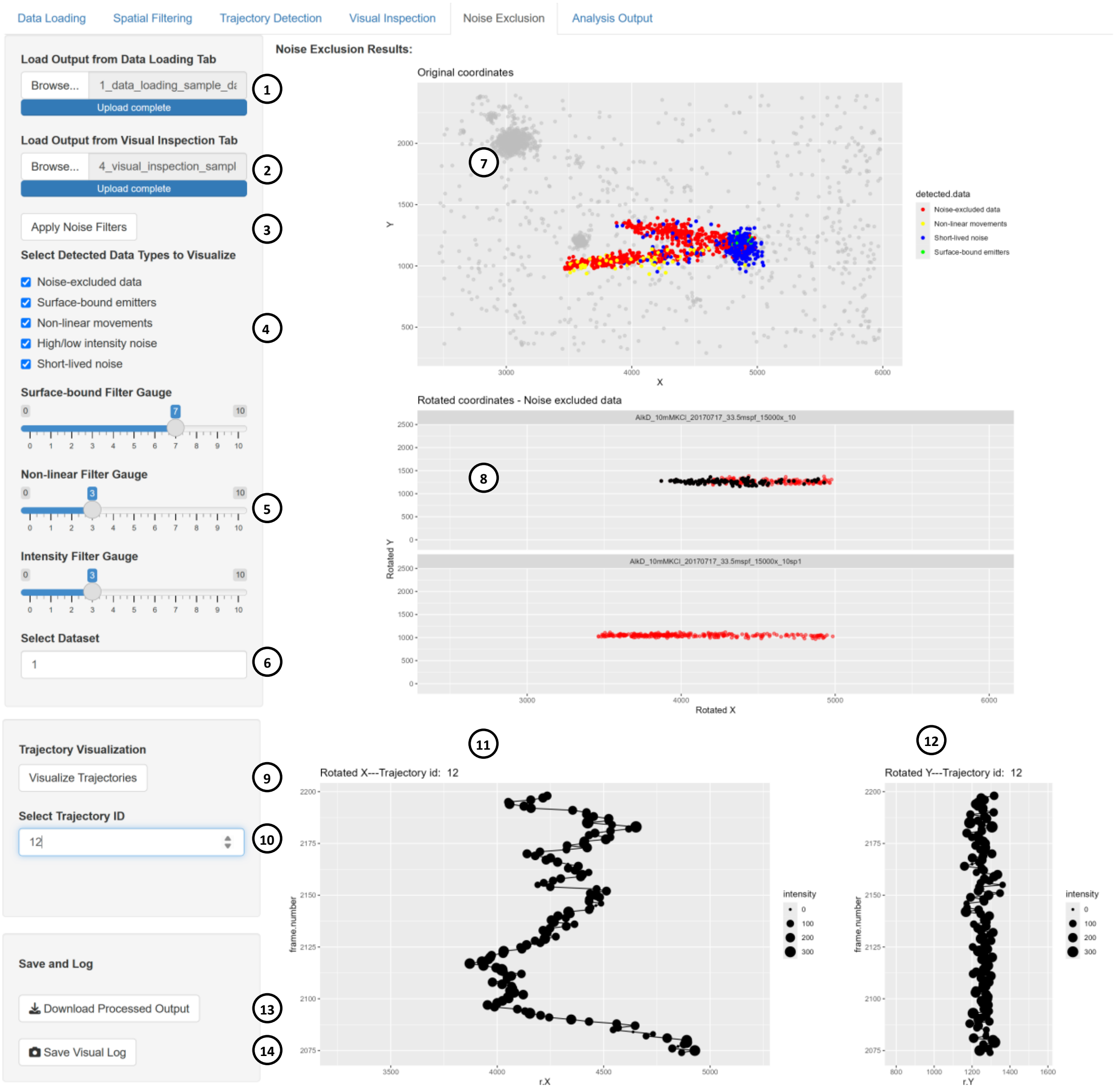

Fig.6 | Fifth module of the SMITracker platform (noise exclusion). In this module, the noise is excluded from the data using the noise exclusion model, and its parameters are defined using the optimization datasets from the previous module. Whatever parameters are selected here are applied to all the datasets homogeneously, and no changes to any particular dataset are possible. It is important to check the performance of the model on all the datasets before finalizing the parameters. Each part of the figure has been marked with numbers corresponding to the steps in the procedure with detailed explanation in the text.

7. Analysis output

Fig.7 shows the first part of the *Analysis Output* module where all the processed data can be visualized. The content of this module is divided into a data overview section and 6 different visualization sections each exploring various parameters to characterize the movement. The numbers in the procedure refer to different parts of the interface as shown in Fig.7.

1. Navigate to files saved from the *Noise Exclusion* module and press the *Add Data* button to load the data.
    a. This step can be repeated for as many datasets as exist, and the data will be accumulated.
2. Include or exclude data from particular proteins in the overview.

3. Sort the overview according to experimental conditions.
4. Explore the table to check the number of datasets, detected trajectories, and frames for each protein and experimental condition.
    a. This data summary can help maintain a statistical balance of the data belonging to each category. Categories with a considerably lower number of trajectories compared to other categories might return less reliable output.
5. Press the *Display Results* button to visualize the results in each section.
6. Choose which protein to include in the visualization.
7. Choose which experimental condition to map onto the color aesthetics of the plot.
    a. Steps 5 to 7 can be repeated for all 6 visualization sections in this module, as described in detail in the Results section of the main text.

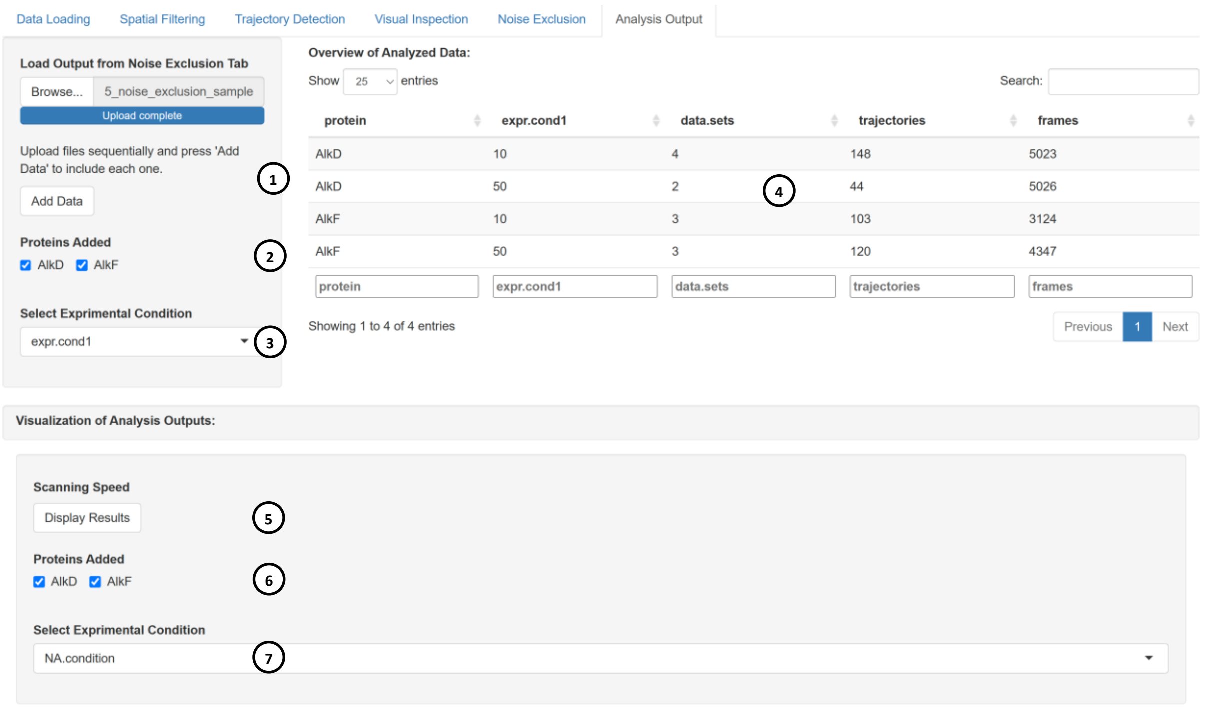

Fig.7 | Sixth module of the SMITracker platform (analysis output, data overview). The first part of the module shows an overview of the analyzed data including the number of datassets, trajectories and observations for each dataset and experimental conditions (numbers 1-4). The second part of the figure (number 5-6) shows a common control panel for visualizations of the results (as described in detail in the Results section of the main text) where the user can select the proteins and experimental conditions for each part of the data visualization in the *Analysis Output* module. Each part of the figure has been marked with numbers corresponding to the steps in the procedure with detailed explanation in the text.
